# Environmentally relevant levels of four psychoactive compounds vary in their effects on freshwater fish condition: a brain concentration evidence approach

**DOI:** 10.7717/peerj.9356

**Published:** 2020-07-09

**Authors:** Pavla Hubená, Pavel Horký, Roman Grabic, Kateřina Grabicová, Ondřej Slavík, Tomáš Randák

**Affiliations:** 1 Department of Zoology and Fisheries, Czech University of Life Sciences Prague, Prague, Czech Republic; 2 Faculty of Fisheries and Protection of Waters, South Bohemian Research Center of Aquaculture and Biodiversity of Hydrocenoses, University of South Bohemia in České Budějovice, Vodňany, Czech Republic

**Keywords:** Food intake, Mortality, Antidepressant, SSRI, Behaviour, Chronic exposure, Growth

## Abstract

**Background:**

The aquatic environment has been contaminated with various anthropogenic pollutants, including psychoactive compounds that may alter the physiology and behavior of free-living organisms. The present study focused on the condition and related mortality of the juvenile chub (*Squalius cephalus*). The aim of the study was to test whether the adverse effects of the antidepressants sertraline and citalopram, the analgesic tramadol and the illicit drug methamphetamine, on fish condition exist under environmentally relevant concentrations and whether these effects persist after a depuration period. Innovative analyses of the fish brain concentrations of these compounds were performed with the aim to show relationship between compound brain tissue concentration and fish condition.

**Methods:**

The laboratory experiment consisted of 42 days of exposure and a subsequent 14-day depuration period with regular monitoring of the condition and mortality of exposed and control fish. Identical methodology, including individual brain concentration analyses for the tested compounds, was applied for all substances. Additional study on feeding under sertraline exposure was also conducted. The feeding was measured from the 28th day of the exposure, three times in a week, by observation of food intake during 15 minutes in social environment.

**Results:**

The effects of particular psychoactive compounds on chub condition varied. While sertraline induced a lower condition and increased mortality, the effects of methamphetamine were inverse, and tramadol and citalopram had no significant effect at all. Individual brain concentrations of the tested compounds showed that the effects of sertraline and methamphetamine on fish condition were increased with brain concentration increases. Additionally, the food intake was reduced in case of sertraline. In contrast, there was no relationship between tramadol and citalopram brain tissue concentration and fish condition, suggesting that the concentration-dependent effect is strongly compound-specific. Methamphetamine was the only compound with a persistent effect after the depuration period. Our results demonstrate the suitability of the brain concentration evidence approach and suggest that changes in fish condition and other related parameters can be expected in freshwater ecosystems polluted with specific psychoactive compounds.

## Introduction

Pharmaceuticals and personal care products (PPCPs) have received significant scientific attention because of their potential environmental fates and effects (Evgenidou, Konstantinou & Lambropoulou, 2015). Generally, drugs for human and veterinary use have been invented to invoke drug-specific reactions in patients’ bodies because of their interaction with drug targets (Corcoran, Winter & Tyler, 2010). The drugs are incompletely degraded in users’ bodies, which leads to their excretion and the subsequent release of various substances into the environment via wastewater (Heberer, 2002). The presence of various drugs in waters has been demonstrated to alter the physiology and behavior of aquatic fauna (Brodin et al., 2013; Gunnarsson et al., 2009; Kümmerer, 2009; Randak et al., 2009; Simmons et al., 2017).

In the present study, we focused on four psychoactive compounds, sertraline, citalopram, tramadol and methamphetamine, which are widely found in water bodies worldwide (Evgenidou, Konstantinou & Lambropoulou, 2015; Fedorova et al., 2014; Kim et al., 2007). Sertraline and citalopram are selective serotonin reuptake inhibitors (SSRIs), which are used as antidepressants in humans. During the initial action of SSRIs, antidepressants in the human brain bind to the serotonin transporter SERT and thus block its function (Hiemke & Härtter, 2000; Rossi et al., 2008). After two to three weeks of treatment, the therapeutic effects manifest as enhanced serotonergic transmission, resulting in alleviation of the symptoms of depression (McDonald, 2017; Murdoch & McTavish, 1992; Noble & Benfield, 1997; Rossi et al., 2008; Stahl, 1998a). Therefore, the acute and chronic treatment effects of SSRIs may vary (Winberg & Thörnqvist, 2016). Sertraline is of a particular interest in this manner, because of its bioaccumulative properties (Grabicova et al., 2017). Specifically, a metabolite of sertraline, norsertraline, reached the highest bioaccumulation factor (it means the ratio of concentration of norsertraline in biota to its concentration in water) within the antidepressants found in fish from natural conditions (Arnnok et al., 2017). In case of Grabicova et al. (2017), which tested the bioaccumulation factor in two different natural sites, the bioaccumulation factor for sertraline in brain was 1500 and 680. It is generally assumed that if the bioaccumulation factor is higher than 500, the compound has tendency to bioaccumulate (according to Organization for Economic Co-operation and Development, 2001). Therefore, some effects of sertraline may be revealed throughout the chronic exposure, during which the concentration of compound in the tissue increases. Tramadol is a drug with a weak affinity for opioid µ-receptors and weak inhibition of norepinephrine and serotonin reuptake (Grond & Sablotzki, 2004; Scott & Perry, 2000). This dual action of tramadol causes pain relief but could also result in antidepressant effects (Barber, 2011; Kalra, Tayal & Chawla, 2008; Markowitz & Patrick, 1998). Methamphetamine (METH) is in low dosages prescribed to treat the attention deficit hyperactivity disorder (Castle et al., 2007); however, it is generally known as an illicit substance with complex action on the central nervous system, which consequently leads to neural damage (Moszczynska & Callan, 2017; Schep, Slaughter & Beasley, 2010). Amphetamines reverse the function of dopamine, noradrenaline, serotonin and vesicular membrane transporters and increase the release of neurotransmitters into the synaptic cleft (Riddle, Fleckenstein & Hanson, 2006; Sulzer et al., 2005). Therefore, neurotransmitters bind to available receptors and intensify transmission. The predicted values of sertraline concentrations in sewage treatment plant effluents range from 0.26 to 3.5 µg/L, and the predicted citalopram concentration ranges from 0.27 to 2.6 µg/L (Christensen et al., 2009). The estimated surface water concentration for tramadol in the Czech Republic ranged from 12 to 210 ng/L, and for methamphetamine, it ranged from 0.82 to 107 ng/L (Fedorova et al., 2014). Generally, the average detected concentrations of the present compounds in surface waters are in range of 24–58 ng/L for citalopram and 240–1400 ng/L for tramadol (Grabicova et al., 2017). The average detected concentration of methamphetamine in surface waters ranged from 13–1800 ng/L (Mackul’ak et al., 2016). Several studies have already reported the effects of pharmaceuticals and illicit drugs on the behavior of fish, such as shelter-seeking in fathead minnow (*Pimephales promelas*; (Valenti Jr et al., 2012) or aggressiveness in Siamese fighting fish (*Betta splendens*; Kania & Wrónska, 2015).

Studies on fish condition assume that fish with a higher weight of a given length are in a better condition (Bolger & Connolly, 1989; Jones, Petrell & Pauly, 1999). Therefore, the condition hints at the ‘well-being’ or ‘fitness’ of individuals. The length and weight of individuals, which are necessary for the calculation of condition, may change with the season, locality or fish age (Froese, 2006). Moreover, growth can also be affected by environmental stressors, such as the presence of pollutants in the environment (Nallani et al., 2016). The possibility of evaluating the well-being of fish has led to the calculation of condition factors in studies and fisheries even today (Brauer et al., 2020; Konoyima et al., 2020).

Pharmaceuticals have been shown to affect food intake in various studies (Hedgespeth, Nilsson & Berglund, 2014; Gröner et al., 2017). However, information about changes in condition in relation to the brain levels of pharmaceuticals of fish has been scarcely assessed, even though changes in length-weight relationships in fish are known to occur in polluted environments (Hubená, Horký & Slavík, 2018). The present study applied a chronic treatment for 42 days to estimate the long-lasting effects of these compounds in polluted natural habitats and focused on the condition (measured from length and weight), brain concentration of the compound of interest and mortality of the juvenile chub (*Squalius cephalus*). The chub is a common omnivorous cyprinid of European rivers (Balestrieri et al., 2006; Krywult, Klich & Szarek-Gwiazda, 2008), that reaches even highly polluted streams, presumably in search of a source of food (Silkina, Mikryakov & Mikryakov, 2016).

The aim of the study was to test: (A) whether the adverse effects of the various psychoactive compounds tested (i.e., sertraline, citalopram, tramadol and methamphetamine) exist under long-term exposure at an environmentally relevant concentration; (B) whether these effects persist after the depuration period (i.e., a pharmaceutical-free period); and (C) whether there is a direct link between the individual brain concentration of the tested compounds and fish condition.

## Materials & Methods

The experiments with particular psychoactive compounds were conducted separately in an identical manner as described in the sections below.

### Experimental animals

The fish used in the experiment were hatchery-reared juvenile chubs obtained from a local fish supplier (Czech Fishery Ltd.; Czech Republic). A total of 320 similarly sized 0+ juvenile fish (five or ten months old for autumn and spring tests, respectively) were transported from the hatchery to the laboratory before testing each particular compound (i.e., A total of 1280 individuals were used for all compounds; the overall average standard length of the fish was 101 ± 13 mm (mean ± S.D.), and the overall average weight of the fish was 7.56 ± 3.46 g (mean ± S.D.). Two weeks prior to the start of the experiment, the fish were divided into four separate holding tanks (200 l with 80 randomly selected individuals each). Half of the identical tanks were intended for the exposed groups, and the second half were intended for the control groups. The fish were fed *ad libitum* on food pellets once a day and kept under a photoperiod of 12 h of light/12 h of darkness, maintaining the same regime to which they were accustomed in the hatchery. Two-thirds of the water volume was renewed with aged dechlorinated municipal tap water every day to habituate the fish to the experimental regime. The water temperature was controlled automatically using air conditioning throughout the whole experiment and held at an average of 20.8 ± 0.4 ^∘^C (mean ± S.D.). Fish health, defined as normal appearance and behavior, including normal body position, movements and food intake (FAO, 1983), was monitored daily. Mortality during the experiment was also noted on a daily basis, but it was not primarily anticipated in the study design because environmentally relevant concentrations of the compounds were used in the present study, aiming to simulate the conditions in real-life polluted waters.

### Pharmaceutical exposure

After acclimatization, fish in the exposed groups were separately exposed to the pollutants at an environmentally relevant concentration of 1 µg/L for 42 days, followed by a depuration period with tested compound-free water until the 56th day (two weeks of depuration). Individual psychoactive compounds (citalopram and sertraline from AK Scientific, Inc., USA, METH and tramadol from Sigma-Aldrich) were dissolved in ultra-pure water (AquaMax Basic 360 Series and Ultra 370 Series instrument, Younglin, purchased from Labicom, CR) to prepare a stock solution at a concentration of 10 mg/L. For the exposure bath, one mL of stock solution was added to every 10 L of aged dechlorinated municipal tap water in the holding tank. All environmental variables (i.e., the temperature, photoperiod and water renewal) were kept the same as those during the acclimatization period (see the Experimental Animals section). The compound was added during the water renewal to keep its concentration in the tanks at the required level. The control fish in the laboratory were kept under the same regime but without the psychoactive compound treatment. The tested compound concentration in the holding tanks was checked every other week during the exposure as well as during the depuration (32 water samples altogether) to verify the real concentrations in the exposed fish and exclude the possibility of cross-contamination in the control groups. The chemical analysis was performed according to Hossain et al. (2019), where isotopically labelled internal standards (citalopram-D_6_, Toronto Research Chemicals; METH-D_5_, Chiron Chemicals; sertraline-D_3_ and tramadol-D_3_, both from Lipomed) were added to filtered water (regenerated cellulose filter, 0.20 µm), and the samples were analyzed by liquid chromatography with tandem mass spectrometry (TSQ Quantiva, Thermo Fisher Scientific) for 10 min on a Hypersil Gold aQ column (50 ×  2.1 mm, 5 µm particles, Thermo Fisher Scientific) with heated electrospray ionization in positive mode (HESI+). The limits of quantification (LOQs) ranged from 0.011 to 0.021 µg/L for citalopram, from 0.010 to 0.012 µg/L for sertraline, from 0.0077 to 0.017 µg/L for methamphetamine, and 0.030 to 0.060 µg/L for tramadol. During the exposure, the average concentration of individual psychoactive compounds in the treated tanks was as follows: citalopram 1.3 ± 0.2 µg/L (mean ± S.D.); sertraline 0.23 ± 0.07 µg/L (mean ± S.D.); methamphetamine 1.1 ± 0.1 µg/L (mean ± S.D.); tramadol 0.99 ± 0.18 µg/L (mean ± S.D.). The concentrations in the tanks of the control fish as well as in the tanks of the exposed fish during depuration were below the limits of quantification.

### Experimental design

The length and weight of eight randomly selected exposed and eight randomly selected control fish (four specimens per tank) were tested after 1, 7, 21 and 42 days of exposure and on the 56th day of the experiment (i.e., the day of exposure and two weeks after depuration). Thus, five trials with 80 specimens were conducted altogether. These specimens were killed according to valid law 246/1992, § 17 and the above-cited permit to analyze the psychoactive compound concentrations in their brains. The brains of freshly killed fish were dissected, weighed and stored frozen at −20 °C for later pharmaceutical content analyses. Before the analyses, the brain samples were defrosted and extracted according to the procedure described in the work of Grabicova et al. (2018). Briefly, isotopically labelled psychoactive compound and extraction solvent were added to approximately 0.1 g of brain tissue. The samples were homogenized, centrifuged, filtered and frozen for 24 h. Then, the extracts were centrifuged again, and the aliquots were analyzed using liquid chromatography with a high-resolution mass spectrometer (Q-Exactive; Thermo Fisher Scientific) on a Hypersil Gold aQ column for seven minutes in HESI+. The LOQs ranged from 0.12 to 0.34 ng/g wet weight (ww) for citalopram, from 0.10 to 0.44 ng/g ww for sertraline, from 0.16 to 0.43 ng/g ww for methamphetamine and from 0.19 to 0.69 ng/g ww for tramadol.

The analyzed concentration of psychoactive compounds was standardized by brain weight (ng/g). Please see Table 1 for details regarding the conditions of the chemical analyses.

**Table 1:** Information about chemical analysis of water and brain samples by liquidchromatography with mass spectrometry.

TSQ Quantiva				Q-Exactive			
Elution gradient for liquid chromatography				Elution gradient for liquid chromatography			
Time [min]	**A** **[%]**	**B** **[%]**	**Flow** **[µL/min]**	**Time** **[min]**	**A** **[%]**	**B** **[%]**	**Flow** **[µL/min]**
0	100	0	300	0	100	0	350
1	100	0	300	1	100	0	350
5	60	40	350	3	50	50	350
7	20	80	400	4	20	80	450
8	0	100	400	5	0	100	450
8.01	100	0	300	6	0	100	450
10	100	0	300	6.05	100	0	350
				7	100	0	350

Mass transitions for mass spectrometry					Mass transitions for mass spectrometry				
Analyte	**Parent ion**	**Quan**	**Qual**	**RT** **[min]**	**Analyte**	**Parent ion**	**Quan**	**Qual**	**RT** **[min]**
citalopram	325.20	109.06	262.07	6.10	citalopram	325.17	109.0449	262.1019	4.10
citalopram-D_6_	331.27	108.825	–	6.10	citalopram-D_6_	331.21	109.0450	–	4.10
methamphetamine	150.11	91.11	119.11	4.14	methamphetamine	150.13	91.0545	119.0854	3.45
methamphetamine-D_5_	155.22	92.11	–	4.14	methamphetamine-D_5_	155.16	92.0611	–	3.45
sertraline	306.06	159.00	274.93	6.75	sertraline	306.08	275.0389	158.9764	4.34
sertraline-D_3_	309.18	159.00	–	6.75	sertraline-D_3_	309.10	158.9764	–	4.38
tramadol	264.23	58.17	246.11	4.80	tramadol	264.20	58.0658	264.1953	3.68
tramadol-D_3_	267.17	58.11	–	4.80	tramadol-D_3_	267.21	58.0658	–	3.68

**Notes.**

A–mobile phase ultra-pure water + 0.1% formic acid; B –mobile phase acetonitrile + 0.1% formic acid.

### Additional experiment (sertraline)

Data on food intake under sertraline exposure were collected regularly from the fourth to eighth week of the experiment from Friday to Sunday (three consecutive days every week, resulting in 15 trials). Food intake was not measured in the first three weeks because of the delayed therapeutic effects of SSRIs, which usually take two to three weeks (Rossi et al., 2008). Feeding experiments were conducted directly in the holding tanks to avoid causing distress to the fish and to prevent the results from being affected by the animal manipulation. Five grams of food pellets (i.e., 1% of the average weight of fish in the particular holding tank; food supply was adjusted according to mortality) were proffered to fish. The presence of non-ingested food in particular tanks was monitored for 15 min in one-minute intervals and noted every minute as a binomial value (0 –not all food pellets were ingested; 1 –all food pellets were ingested). A limit of 15 min was established based on the preliminary testing of food intake during regular feeding, which lasted from seven to eight minutes (i.e., the limit was double the regular feeding time).

### Statistical analyses

‘Treatment’ was defined as the class variable distinguishing whether fish were exposed to sertraline. ‘Experimental phase’ distinguished between the exposure and depuration phases. ‘Compound in brain tissue’ expressed the relative concentration of particular psychoactive compound in the individual fish brain tissue. Fulton’s condition factor ‘K’ was evaluated as K = M/LS3, where M is mass and LS is the standard length. The compound concentration in brain tissue and Fulton’s condition factor were assigned for every sampled individual fish.

‘Food ingestion’ was assigned only for sertraline treatment as a count variable expressing number of one-minute intervals per feeding trial until all the food was eaten. Thus, it could acquire values from 1 to 15 (tanks where the all food was not eaten got a maximum score of 15 out of the 15 intervals). ‘Mortality’ was assigned as a count variable expressing a number of dead individuals in a particular holding tank per day. Food ingestion and mortality were assigned for every tank.

The statistical analyses were performed using the SAS software package (SAS Institute Inc., version 9.4, http://www.sas.com). The effects of all four psychoactive compounds (i.e., sertraline, citalopram, tramadol and methamphetamine) were tested separately.

Differences between Fulton’s condition factor ‘K’ of control and exposed fish and progress of ‘brain compound concentration’ of exposed fish during particular time samples (i.e., 1, 7, 21, 42, 56th day of the experiment) were determined with a separate *t*-tests and presented as boxplots of raw data. Generalized linear mixed models (GLMM; SAS function PROC GLIMMIX) with a Poisson distribution were used to analyze the variables ‘food ingestion’ and ‘mortality’. Particular holding tank and date of sampling were used as class random factors for both dependent variables. As feeding could be hypothetically influenced by fish weight, we included mean weight and its standard deviation obtained from individual condition measures as continuous random factors for analyses of ‘food ingestion’ as well. A separate model for each dependent variable was fitted, using interaction of ‘treatment’, and ‘experimental phase’ as the class explanatory variable. Generalized linear mixed model (GLMM; SAS function PROC MIXED) with a normal distribution was used to analyze Fulton’s condition factor ‘K’. Individual fish and date of sampling were used as a class random factors. Two models for dependent variable ‘K’ were fitted due to the ‘treatment’ and ‘compound in brain tissue’ variables overlap. Thus, first model contained ‘treatment’ fixed factor variable, while second model included ‘compound in brain tissue’ fixed factor variable. The significance of the exploratory variables was assessed using an *F*-test. Least-squares means (henceforth referred to and in bar charts presented as ‘adjusted means’ of model predictions) were subsequently computed for the particular classes of class variables. Differences between the classes were determined with a *t*-test, and a Tukey–Kramer adjustment was used for multiple comparisons. Association between the dependent variables ‘K’ and other continuous variable ‘compound in brain tissue’ was estimated by fitting a random factor model using PROC MIXED as described by Tao et al. (2002). With this random coefficient model, we calculated the predicted values for the dependent variable ‘K’ and plotted them against the continuous variable ‘compound in brain tissue’ by using the predicted regression lines in particular scatter charts. The degree of freedom was calculated using the Kenward–Roger method (Kenward & Roger, 1997).

### Ethical note

All laboratory experimental procedures complied with valid legislative regulations (law no. 246/1992, § 19, art. 1, letter c) and were carried out with the relevant permission from the Ministry of Education, Youth and Sports of the Czech Republic (permit no. MSMT-1972/2016-5, registered by the Ministry of Education, Youth and Sports of the Czech Republic). The Departmental Expert Committee supervised the animal welfare in the present study. The research staff was trained according to valid legislative regulations on laboratory animal care, handling and welfare. The number of fish used corresponded to the reduction in laboratory animals used, and the use of animals could not be replaced by any other known method to obtain the relevant data.

## Results

### Condition factor progression

**Figure 1:**
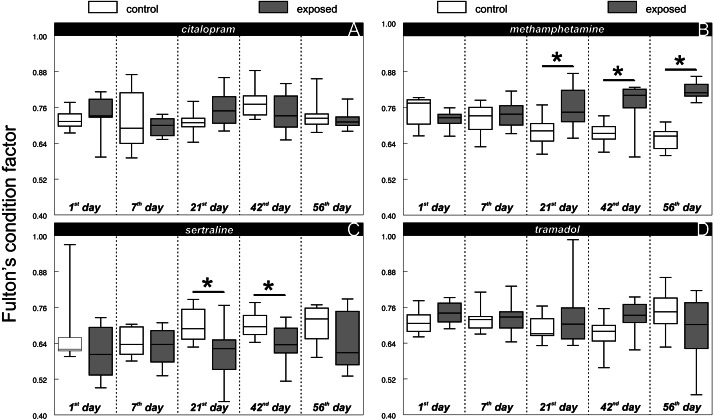
Fulton’s condition factor ‘K’ of control and exposed fish during particular time samples (i.e., 1, 7, 21, 42, 56th day of the experiment) across citalopram (A), methamphetamine (B), sertraline (C) and tramadol (D) experiment. Boxplot with whiskers from minimum to maximum originate from raw data. Asterisks indicate significant differences.

**Figure 2:**
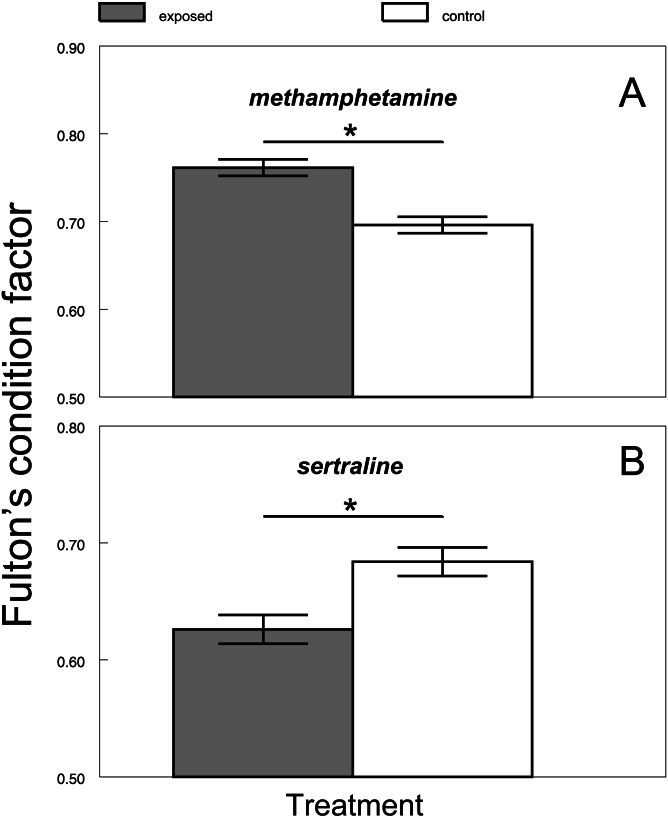
Differences between predicted Fulton’s condition factor ‘K’ of control and exposed fish across methamphetamine (A) and sertraline (B) experiment. Values are adjusted means ± SE predicted from particular mixed models. Asterisks indicate significant differences. Figure covers the time span of whole experiment (i.e., exposure and depuration together).

There was no difference between the chub condition under the control and exposure treatments at the beginning of all experiments (1st day: citalopram, *t* = 0.71, *P* > 0.48, *n* = 16; Fig. 1A; methamphetamine *t* = 1.33, *P* > 0.2, *n* = 16; Fig. 1B; sertraline, *t* = 1.09, *P* > 0.29, *n* = 16; Fig. 1C; tramadol, *t* = 1.59, *P* > 0.14, *n* = 16; Fig. 1D). Citalopram (F_1,78_ = 0.01, *P* > 0.93) and tramadol (F_1,78_ = 0.96, *P* > 0.33) treatment did not cause any overall changes in chub condition when compared to the control. Additionally, comparison of condition during particular days did not show any significant differences (Fig. 1A, citalopram: 7th day, *t* = 0.57, *P* > 0.58, *n* = 16; 21st day, *t* = 1.79, *P* > 0.09, *n* = 16; 42nd day, *t* = 1.15, *P* > 0.27, *n* = 16; 56th day, *t* = 0.66, *P* > 0.52, *n* = 16; Fig. 1C, tramadol: 7th day, *t* = 0.21, *P* > 0.84, *n* = 16; 21st day, *t* = 0.96, *P* > 0.35, *n* = 16; 42nd day, *t* = 2.09, *P* > 0.06, *n* = 16; 56th day, *t* = 1.24, *P* > 0.23, *n* = 16). In contrast, methamphetamine (F_1,78_ = 24.58, *P* < 0.0001; Fig. 2A) and sertraline (F_1,80_ = 11.15, *P* < 0.0013; Fig. 2B) treatment induced significant overall condition alterations. Methamphetamine caused a condition increase (Adj. *P* < 0.0001; Fig. 2A), which was detectable from the 21st day of exposure (7th day, *t* = 0.64, *P* > 0.54, *n* = 16; 21st day, *t* = 2.52, *P* < 0.03, *n* = 16; 42nd day, *t* = 3.26, *P* < 0.01, *n* = 16) and persisted after the depuration phase (56th day, *t* = 9.51, *P* < 0.01, *n* = 16). In contrast, sertraline caused a decrease in condition (Adj. *P* < 0.0013; Fig. 2B), which was detectable from the 21st day of exposure (7th day, *t* = 0.54, *P* > 0.6, *n* = 16; 21st day, *t* = 2.31, *P* < 0.04, *n* = 16; 42nd day, *t* = 2.2, *P* < 0.04, *n* = 16) and disappeared during the depuration phase (56th day, *t* = 1.38, *P* > 0.19, *n* = 16).

### Psychoactive substances in the fish brain

**Figure 3:**
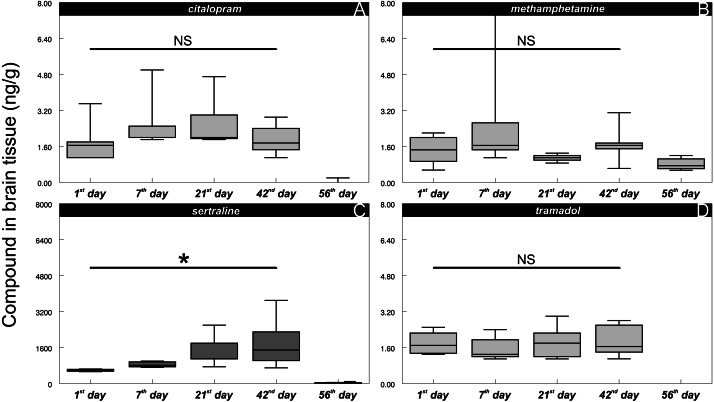
Citalopram (A), methamphetamine (B), sertraline (C) and tramadol (D) in brain tissue during particular time samples (i.e., 1, 7, 21, 42, 56th day of the experiment). Boxplot with whiskers from minimum to maximum originate from raw data. Asterisks indicate significant differences.

In all the control fish, particular psychoactive substances in the brain tissue were below the LOQ throughout the whole experiment, confirming that the control fish were unaffected in this respect. In contrast, fish exposed to environmentally relevant concentrations of psychoactive substances showed positive brain concentrations from the 1st day of exposure (Fig. 3A, citalopram: 1.7 ± 0.79 ng/g (mean ± S.D.); Fig. 3B, methamphetamine: 1.4 ± 0.62 ng/g (mean ± S.D.), Fig. 3C, sertraline: 590 ± 47 ng/g (mean ± S.D.); Fig. 3D, tramadol: 1.8 ± 0.78 ng/g (mean ± S.D.)). Citalopram, methamphetamine and tramadol brain tissue concentrations did not show any clear exposure-related progression, and they did not differ between the 1st and 42nd days of exposure (citalopram:, *t* = 0.53, *P* > 0.6, *n* = 16; methamphetamine: *t* = 0.45, *P* > 0.46, *n* = 16; tramadol: *t* = 0.34, *P* > 0.74, *n* = 16). After the depuration period (56th day), the brain concentration of tramadol was completely below the limit of quantification, and a positive response of citalopram was observed in only one of the eight individuals tested (mean 0.025 ± 0.070 ng/g (mean ± S.D.)), but methamphetamine was still detectable in all sampled fish (mean 0.82 ± 0.25 ng/g (mean ± S.D.)). In contrast to other compounds tested, the sertraline mean concentration in the exposed fish brain tissue subsequently increased during exposure (1st day—mean 590 ± 47 ng/g (mean ± S.D.); 7th day—mean 850 ± 120 ng/g (mean ± S.D.); 21st day—mean 1400 ± 660 ng/g (mean ± S.D.); 42nd day—mean 1800 ± 1000 ng/g (mean ± S.D.)) with significant differences between the 1st and 42nd days of exposure (*t* = 3.25, *P* < 0.01, *n* = 16). Despite this fact, the drug uptake was strongly individually driven as there were specimens reaching concentrations around 700 ng/g throughout the experiment. Low but still detectable levels of sertraline were also observed in all fish after depuration (56th day—mean 32 ± 17 ng/g (mean ± S.D.).

**Figure 4:**
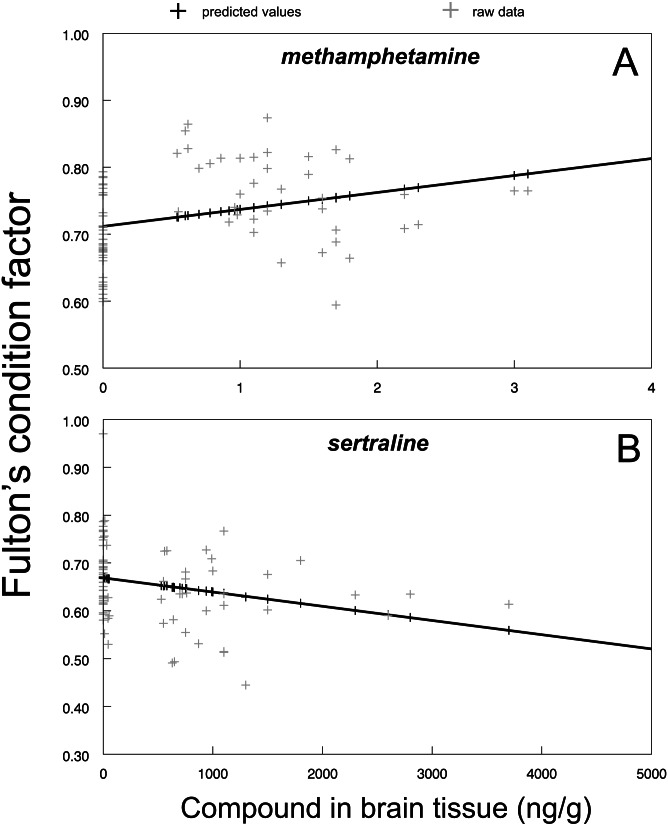
Fulton’s condition factor ‘K’ in relation to methamphetamine (A; predicted regression line is fitted by *y* = 0.7116 + 0.0253*x*; *r*^2^ = 0.9) and sertraline (B; predicted regression line is fitted by *y* = 0.6688 − 0.0001*x*; *r*^2^ = 0.9) in brain tissue. Predicted values from particular mixed model are shown as dark markers, raw data are shown as shaded markers.

The direct link between methamphetamine (F_1,77_ = 7.71, *P* < 0.0069; Fig. 4A) and sertraline (F_1,80_ = 5.86, *P* < 0.0178; Fig. 4B) brain tissue concentrations and individual chub condition indicated that there is a strong influence of the individual amount of the specific drug received from the environmentally relevant water concentrations. The effect of methamphetamine and sertraline was opposite: increased methamphetamine concentrations were related to higher chub conditions (Fig. 4A) and vice versa for sertraline (Fig. 4B). In contrast, there was no significant relationship between tramadol (F_1,78_ = 3.47, *P* > 0.07) and citalopram (F_1,78_ = 0.00, *P* > 0.96) brain tissue concentration and fish condition, suggesting that the concentration-dependent effect was strongly compound-specific.

### Mortality

**Figure 5:**
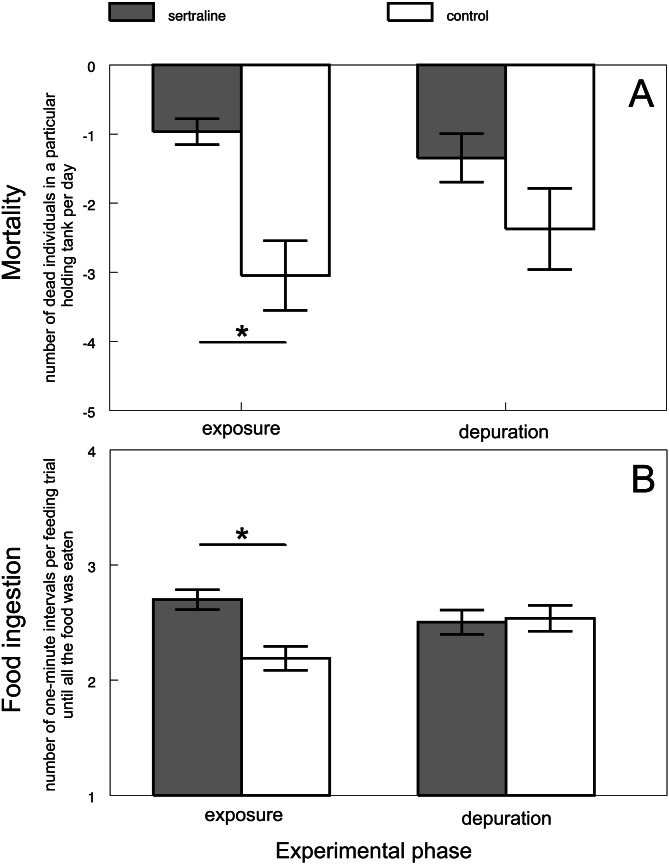
Predicted values of mortality (A) and food ingestion (B) in relation to experimental phase and treatment of sertraline experiment. Values are adjusted means ± SE predicted from particular mixed models. Asterisks indicate significant differences.

Mortality varied across all control and most treatment groups (i.e., tramadol, citalopram and methamphetamine) from 0 to 8 individuals (i.e., 0–5%) throughout the whole experiment lasting 56 days. There was never found more than 1 dead individual during the daily controls. Dead fish showed no obvious signs of disease- or condition-related problems. In contrast, sertraline treatment caused mortality in 42 individuals (i.e., 26%). Up to 4 dead individuals were found during daily controls, and dead fish showed signs of lowered condition. The increased mortality of sertraline-treated fish was significantly higher than that of the control fish during exposure (F_3,230_ = 6.22, *P* < 0.0004; Adj. *P* < 0.0008 Fig. 5A). This adverse effect disappeared during the depuration phase (Adj. *P* > 0.44).

### Feeding under sertraline exposure (additional experiment)

Sertraline affected the feeding behavior of chub during the exposure phase. The food ingestion was lower in the exposed fish than in the control fish (F_3,8.571_ = 5.49, *P* < 0.0218; Adj. *P* < 0.0206; Fig. 5B). In other words, sertraline caused a decrease in food consumption. The adverse effects disappeared during the depuration phase (Adj. *P* > 0.99).

## Discussion

In the present study, we aimed to simulate the long-term exposure of fish to various psychoactive compounds. The effects of environmentally relevant levels of these compounds varied from a decrease in condition and related adverse effects in the case of sertraline to an increase in condition in the case of methamphetamine to non-detectable effects in case of citalopram and tramadol. Various impacts of particular compounds may reflect their different modes of action (Stahl, 1998b) as well as species-specific effects. For instance, chub in the present study did not show a significant change in condition under the environmental concentration of citalopram, while the opposite was shown in the three-spine stickleback (*Gasterosteus aculeatus*; Kellner et al., 2015). The condition effects, however, were also individually dependent, as was shown by our progressive brain concentration analyses. We found a direct link between individual brain concentrations of sertraline and methamphetamine and the degree of its influence on chub condition. Variations in individual compound consumption at the same aquatic concentration are presumably the result of personality (Manning & Dawkins, 2012) and related individual metabolic rates (Metcalfe, Van Leeuwen & Killen, 2016). If we adopt this concept, then particular individuals from the same polluted aquatic environment face different pressures based on their personality, which could influence the makeup of populations (Brown et al., 2009) based on which types of individuals are likely to be preferred if psychoactive substances alter their performance.

The sertraline concentration in the brain was negatively correlated with chub condition in the present study. The sertraline mechanism of action causes prolonged activation of the serotonergic system (Murdoch & McTavish, 1992). Similar heightened activity of the serotonergic system has been found in subordinate individuals in species such as rainbow trout (*Oncorhynchus mykiss*), Arctic charr (*Salvelinus alpinus*) and Atlantic salmon (*Salmo salar*; Alanärä et al., 1998; Cubitt et al., 2008; Øverli et al., 1998; Vindas et al., 2016; Winberg et al., 1993; Winberg & Nilsson, 1993). These studies have demonstrated that subordinates also reduce food intake and have lower growth rates than dominant fish characterized by lower serotonergic activity (Alanärä et al., 1998; Cubitt et al., 2008; Øverli et al., 1998; Vindas et al., 2016; Winberg et al., 1993; Winberg & Nilsson, 1993). Sertraline has been demonstrated to decrease food intake, but only at concentrations higher than the environmental levels (Hedgespeth, Nilsson & Berglund, 2014; Chen, Gong & Kelly, 2017; Xie et al., 2015). The environmental concentration of sertraline has been tested previously for six to eight days only (Hedgespeth, Nilsson & Berglund, 2014; Chen, Gong & Kelly, 2017). We observed a significant decrease in food intake in chub, which was measured from the 28th day of the supplementary experiment, following the change in the condition that appeared from the 21st day. Thus, we suggest that future experiments should consider the first three weeks of sertraline exposure separately, as the effects during this time might not represent the chronic effects of this compound because of the adaptive change in the serotonergic system after two to three weeks of treatment (Cowen & Sargent, 1997). Therefore, fish chronically exposed to sertraline could possibly have impaired energy intake through lower food ingestion. In the present study, we assume from the calculation of Fulton’s condition factor that the exposed fish gained less weight at a given length than control individuals. Fulton’s condition factor is also thought to be a good indicator of fish resistance to chemical pollution or stress (Owen et al., 2009). Simultaneously, with lower resistance and energy storage, the fish might struggle with survival, as was proven in the present study, with a mortality of 26% in the exposure groups under environmental concentrations. We note that mortality following exposure to environmental concentrations of 5.2 ng/L of sertraline was observed even in fathead minnow (Schultz et al., 2011). Schultz et al. (2011) warned of the chronic exposure effects of this compound because of its bioaccumulative properties. A plethora of studies have found a significant bioaccumulation of sertraline in tissues (Chen, Gong & Kelly, 2017; Grabicova et al., 2017; Koba et al., 2018; Xie et al., 2015; Grabicova et al., 2014). Specifically, sertraline was found in the liver and other tissues, including the brain, kidneys, gills and muscles (Xie et al., 2015; Grabicova et al., 2017). The increasing levels of sertraline in the brain in the present study support these findings.

The sertraline-free period resulted in compensatory growth of chub because no condition changes were detectable, even though a low sertraline brain concentration was still detected. Compensatory growth has also been demonstrated in other fish species, such as hybrid striped bass (Skalski et al., 2005), three-spined stickleback (*G. aculeatus*; Inness & Metcalfe, 2008) and gibel carp (*Carassius auratus gibelio*; Xie et al., 2001). The growth after food deprivation in barramundi (*Lates calcarifer*) was dependent on the duration of food deprivation; thus, it may have resulted in partial compensation only (Tian & Qin, 2003). However, complete compensation was detected after six weeks of sertraline exposure followed by two weeks of depuration. Despite the fact that different situations could arise over a lifetime of exposure in polluted natural habitats, these results are promising for remediation measures aimed at decreasing pollution in freshwaters.

Chronic methamphetamine exposure resulted in increased chub conditions in the present study. In contrast, goldfish (*Carassius auratus*) injected with amphetamine showed a decrease in food intake and feeding behavior at high concentrations of 25 to 75 µg/g (Volkoff, 2013). Decreases in weight and food intake were also observed in rats injected with methamphetamine, but only during the first days of exposure (Hsieh et al., 2006; Kuo, 2003). The contradictory evidence of methamphetamine may be explained by the concentration and length of the exposure dependency. For instance, Irons et al. (2010) described an increase in locomotion of zebrafish exposed to 0.2 to 6.6 µM amphetamine but a decrease in locomotion at 20 µM. Additionally, most of the studies on fish and rats focus on exposure for only seven days (Crowley et al., 2005; Hsieh et al., 2006; Kuo, 2003; Liao et al., 2015; Zheng et al., 2014). Therefore, after demonstrating a significant change in the condition of fish after 21 days, we promote consideration of the length of the exposure in experiments with environmental concentrations of methamphetamine. Methamphetamine has a complex mode of action in organisms, acting on various substances and neurotransmitters. Crowley et al. (2005) found that rats fed *ad libitum* under methamphetamine exposure showed increased levels of neuropeptide Y in their brains. Neuropeptide Y has been found to stimulate the growth of abdominal fat in rats (Kuo et al., 2007). If we adopt this explanation, the stimulated growth of abdominal fat could explain the higher condition of chub in the present study. Furthermore, we proved that the methamphetamine brain concentration and altered condition persisted after the depuration period. Despite the fact that a withdrawal period in rats has resulted in a significant restoration of metabolic processes, the levels of specific compounds have not returned to normal (Zheng et al., 2014). Specifically, there were altered levels of isoleucine, palmitic acid or compounds involved in the tricarboxylic acid cycle, which has a connection to energy stores and lipid metabolism (Zheng et al., 2014).

Neither citalopram nor tramadol changed the condition of chub in the present study. Despite this fact, we should be cautious in this respect, as all pollutants in the environment should be assumed to be stressors (Nallani et al., 2016), and the effect of psychoactive compounds could be species (Kellner et al., 2015) as well as context specific (Conners et al., 2009; Weinberger II & Klaper, 2014; Dorelle et al., 2020). Nonetheless, in the present study, we demonstrate that not all chemical compounds at environmental concentrations influence the condition factors of fish.

## Conclusions

The present study demonstrates that particular psychoactive compounds in environmental doses may have a variable effect on fish condition during long-term exposure. Most adverse effects were detected for sertraline, as its effect on condition accompanied by disrupted food intake resulted in elevated mortality. Therefore, ecosystems polluted with this bioaccumulative SSRI should be managed with special attention. Our data from innovative brain concentration analyses indicate that particular individuals from the same polluted aquatic environment face different pressures, presumably as a result of their various personalities. Further research should be conducted in this respect to reveal the harmful potential of this phenomenon for the makeup of wild populations. We also highlight the need to consider the time-dependent modes of action and metabolic rates of compounds during establishing the lengths of experimental exposures because some effects may be detectable only after a certain amount of time (e.g., 21 days for sertraline and methamphetamine in our case).

##  Supplemental Information

10.7717/peerj.9356/supp-1 Supplemental Information 1 Fulton’s condition factor in relation to the methamphetamine exposure

10.7717/peerj.9356/supp-2 Supplemental Information 2 Fulton’s condition factor in relation to the citalopram exposure

10.7717/peerj.9356/supp-3 Supplemental Information 3 Fulton’s condition factor in relation to the sertraline exposure

10.7717/peerj.9356/supp-4 Supplemental Information 4 Fulton’s condition factor in relation to the tramadol exposure

10.7717/peerj.9356/supp-5 Supplemental Information 5 Mortality data in relation to the sertraline exposure

10.7717/peerj.9356/supp-6 Supplemental Information 6 Food intake data in relation to the sertraline exposure

## Additional Information and Declarations

### Data Availability

The following information was supplied regarding data availability: The raw measurements are available in the Supplementary Files.
